# Financial Health Among Louisiana Medicaid Enrollees

**DOI:** 10.1001/jamahealthforum.2024.3028

**Published:** 2024-10-11

**Authors:** Chris Frenier, Beniamino Green, Jacob Wallace, Alexander Siebert, Andrew Anderson, Kevin Callison, Brigham Cody Walker

**Affiliations:** 1 Yale School of Public Health, New Haven, Connecticut; 2 Tulane University School of Public Health and Tropical Medicine, New Orleans, Louisiana; 3 Abt Global Inc, Rockville, Maryland; 4 Johns Hopkins Bloomberg School of Public Health, Baltimore, Maryland; 5 Murphy Institute for Political Economy, New Orleans, Louisiana

## Abstract

This cross-sectional study compares average credit scores and unpaid medical and nonmedical debts in collections among Black, Hispanic, and White adults with Medicaid coverage.

## Introduction

Financial instability limits access to medical care and is associated with poor health outcomes, potentially burdensome medical bills, and lower earnings.^1,2^ Financial health also plays a role in housing access, stable employment, and good credit, which are key social determinants of health.^3^ Physical health disparities among Medicaid beneficiaries are well documented,^4,5^ but less is known about financial disparities in this population. This study used novel data to describe the financial health of Medicaid enrollees in Louisiana, specifically Hispanic, non-Hispanic Black (hereafter, Black), and non-Hispanic White (hereafter, White) beneficiaries.

## Methods

We obtained Medicaid enrollment data for March 2018 through December 2019 from the Louisiana Department of Health, which were linked to deidentified credit report data from Equifax Information Services. The sample comprised adults aged 18 to 64 years with continuous Medicaid enrollment for at least 24 months (July 2016-December 2019). The Tulane University and Louisiana Department of Health Institutional Review Boards approved this cohort study and waived informed consent because deidentified data were used. We followed the STROBE reporting guideline.

We constructed 3 primary person–level credit outcomes: average credit score, average monthly balance of unpaid medical debt in collections, and average monthly balance of nonmedical debt in collections. Average monthly balances of medical and nonmedical debt in collections included observations with no debt in collections. We defined poor credit as an average credit score below 580 and assessed proportions of enrollees with any medical or nonmedical debt in collections.^6^ We weighted all analyses to be representative of adults younger than 65 years enrolled in Louisiana Medicaid.

Enrollment data included age, sex, self-reported race and ethnicity, and parish of residence. We reported weighted, unadjusted outcomes and demographic characteristics for Black, Hispanic (all races), and White enrollees. We used linear regression to compare mean values of outcome variables between race and ethnicity subgroups, adjusting for age, sex, and parish of residence.

Two-sided *P* < .05 indicated statistical significance. Analyses were performed between February 2023 and July 2024 using R 4.1.1 (R Core Team). The eMethods in Supplement 1 provides more details.

## Results

The weighted sample included 265 258 individuals, of whom 173 337 were females (65%), 91 920 males (35%) with a mean (SD) age of 37 (14) years. Hispanic enrollees had a younger mean age (34 vs 38 years) and were more likely to be female (73% vs 64%) than White enrollees (Table 1).

**Table 1.** Demographic Characteristics, by Race and Ethnicity^a^

Characteristic	Medicaid enrollees, No. (%)		
	Black (n = 130 643)	Hispanic (n = 22 495)	White (n = 112 120)
Age, mean (SD), y	37 (14)	34 (12)	38 (14)
*P* value^b^	<.001	<.001	NA
Sex			
Female	85 132 (65)	16 364 (73)	71 841 (64)
Male	45 510 (35)	6131 (27)	40 279 (36)
*P* value^b^	.008	<.001	NA
Rurality^c^			
Nonurban	10 049 (80)	18 572 (83)	94 233 (84)
Urban	25 593 (20)	3923 (17)	17 887 (16)
*P* value^b^	.64	.77	NA

Abbreviation: NA, not applicable.

^a^ Demographics observed in Medicaid enrollment data. Means, SDs, and counts reflect the weighted sample but are not adjusted for covariates. The denominator for the percentage outcomes is all enrollees in the race and ethnicity group.

^b^ *P* values for age and sex were obtained from a linear regression of the characteristic on categorical variables for Black and Hispanic race and ethnicity (reference group: White race) with cluster-robust SEs at the parish-level. *P* values for rurality were obtained from a similar regression.

^c^ Urban and nonurban are defined based on parish of residence.

Average credit score was 547 for Black enrollees and 571 for Hispanic enrollees, both are significantly lower than 588 for White enrollees (*P* < .001). Proportion of Black and Hispanic enrollees with poor credit was significantly higher than the proportion of White enrollees (61% and 50% vs 44%; *P* < .001) (Table 2).

**Table 2.** Average Credit Outcomes by Race and Ethnicity^a^

	Medicaid enrollees, No. (%)		
	Black (n = 130 643)	Hispanic (n = 22 495)	White (n = 112 120)
Credit outcomes			
Credit score, mean (SD)	547 (66)	571 (83)	588 (81)
*P* value^b^	<.001	<.001	NA
Poor credit score: <580	79 534 (61)	11 226 (50)	49 363 (44)
*P* value^b^	<.001	<.001	NA
Missing credit score	25 386 (19)	4434 (20)	22 634 (20)
Medical debt outcomes			
Any medical debt in collections	61 325 (47)	10 146 (45)	50 942 (45)
*P* value^b^	<.001	<.001	NA
Medical debt in collections, mean (SD), $	1260 (4412)	1253 (5102)	$1618 (6475)
*P* value^b^	.58	.62	NA
Missing medical debt outcome	24 235 (19)	4261 (19)	21 601 (19)
Nonmedical debt outcomes			
Any nonmedical debt in collections	65 589 (50)	9546 (42)	39 973 (36)
*P* value^b^	<.001	<.001	NA
Nonmedical debt in collections, mean (SD), $	680 (1583)	648 (1608)	$550 (1720)
*P* value^b^	<.001	<.001	NA
Missing nonmedical debt outcome	24 235 (19)	4261 (19)	21 601 (19)

Abbreviation: NA, not applicable.

^a^ Credit outcomes observed in Equifax credit report data. Means, SDs, and counts reflect the weighted sample but are not adjusted for covariates. The denominator for the percentage outcomes is all enrollees in the race and ethnicity group, including those with missing outcomes.

^b^ *P* values were obtained from a linear regression of the outcome on categorical variables for Black and Hispanic race and ethnicity (reference group: White race) with controls for age (linear and quadratic), sex, fixed effects for parish of residence, and cluster-robust SEs at the parish level.

The proportion of enrollees with medical debt in collections was slightly higher among Black and Hispanic enrollees than White enrollees (47% and 45% vs 45%; *P* < .001). Average balance of medical debt in collections did not differ significantly across enrollees.

Average balances of nonmedical debt in collections were higher among Black and Hispanic enrollees than White enrollees ($680 and $648 vs $550; *P* < .001). Proportion of any nonmedical debt in collections was also higher for Black and Hispanic enrollees than White enrollees (50% and 42% vs 36%; *P* < .001).

## Discussion

We found racial disparities in credit scores and nonmedical debt in collections among Louisiana Medicaid enrollees. Black and Hispanic enrollees had significantly worse credit scores and higher burdens of nonmedical debt in collections than White enrollees. Differences in the prevalence and balance of medical debt in collections were either small or not significant.

These findings suggest Medicaid coverage may play a protective role that ameliorates race- and ethnicity-based disparities in financial outcomes associated with medical care. A study limitation was our inability to observe whether Medicaid directly affected credit and debt outcomes or whether disparities in financial health preceded enrollment in Medicaid.

## Notes

Supplement 1.
eMethods.

Supplement 2.
Data Sharing Statement
