# Supplementary material for: Financial Health Among Louisiana Medicaid Enrollees
Source: JAMA Health Forum. 2024 Oct 11;5(10):e243028. doi: 10.1001/jamahealthforum.2024.3028 (PMC11470384; doi:10.1001/jamahealthforum.2024.3028)
Supplement: Supplement 1. — eMethods. [file jamahealthforum-e243028-s001.pdf]

Supplemental Online Content

Frenier C, Green B, Wallace J, et al. Financial health among Louisiana Medicaid enrollees. *JAMA Health Forum.* Published online October 11, 2024. doi:10.1001/jamahealthforum.2024.3028

eMethods.

This supplemental material has been provided by the authors to give readers additional information about their work.

© 2024 Frenier C et al. *JAMA Health Forum*.

eMethods

The data for this study were acquired as part of a larger research project linking Louisiana Medicaid enrollees to credit reporting data from Equifax. The sample of linked data included individuals between the ages of 18 and 64 who were enrolled in Louisiana's Medicaid program for 24 continuous months at any point between July 2016 and December 2019. Due to cost limitations in acquiring linked credit report data from Equifax, the sample strategically over-sampled some parts of the LA Medicaid population. Specifically, the sample includes all enrollees who underwent one of 5 major health events (heart attack, cancer, delivery, concussion, stroke) during the period from July 2016 through December 2019. Identification of enrollees for this population was done using health care claims data to identify the presence and timing of the different health shocks. Members of the study team also submitted a random sample of enrollees who met the age and continuous enrollment criteria, but who did not have one of the five health shocks during the study period, to Equifax for linkage.

Equifax searched this database for credit report records matching the identifiers from the Louisiana Department of Health (LDH) Medicaid data. When a match was identified, Equifax returned monthly credit reporting data variables for the enrollee for each month in the study period where data were available. The final file used by our team was a person-month panel of linked Medicaid-Equifax data. The linkage rate was high, with 81.34 percent of individuals in the LDH Medicaid data finding a match in the Equifax data. In addition, 94.62 percent of the linked individuals are identifiable as either non-Hispanic Black, Hispanic, or non-Hispanic White. All observations in this final file were successfully linked but may been missing credit reporting data for some months during the study period.

During the course of our data analysis, we observed that there were large discontinuities in some of our financial outcomes in early 2018. Discussions with Equifax suggested that this may have occurred because debt records were updated to reflect forgiveness and the clearing of stale debt. To avoid potential bias from these stale or forgiven debts, we restricted our analysis to person-month observations between March 2018 and December 2019.

The demographic data reported in the manuscript come from the LDH data. Age, sex, and parish of residence are based on data from the enrollment database. Our data did not include information about Medicaid enrollment type, but regulations in Louisiana during this period mean that nearly all members of our sample were likely enrolled in a Medicaid managed care plan.

The LDH database contains three variables concerning race and ethnicity: (1) beneficiary ethnicity, (2) beneficiary race at enrollment, and (3) beneficiary race at the claim level. We combined all three to identify Hispanic, non-Hispanic White, and non-Hispanic Black beneficiaries. Race at enrollment was given precedence over the claims-based values. If race at enrollment was missing, race at the claim-level was included. If there was disagreement across claims, the first value for race was utilized.The unit of analysis for this research was the enrollee. We collapsed the person-month-level data to the person-level, using the following rules. For categorical variables (sex, race/ethnicity, rurality) we took the modal value of the variable, breaking ties by selecting the most recent value recorded in the data. For the "any medical debt in collections" and "any non-medical debt in collections" outcomes, we set to variable equal to one if any of the person-months in sample had a non-zero balance of medical or non-medical debt in collections respectively. For continuous variables (credit score, medical debt in collections debt, non-medical debt in collections, age) we calculated the mean for each person across all available months, ignoring missing observations. We coded the "poor credit score" outcome equal to one if the person's mean credit score over the study period was less than 580, which Equifax defines as the threshold for "poor credit."

To account for uneven selection probabilities, we used sample weights to re-weight our analyses to be reflective of the original population of Louisiana Medicaid enrollees who met the age and continuous enrollment requirements. This approach corrects for the oversampling of enrollees that undergo health shocks. The sample weights are equal to 1 if a unit underwent a health shock or 3.919 otherwise. This means that non-shocked individuals are given approximately 4x as much weight as shocked individuals when estimating average outcomes in our analysis. We note again that the non-shocked individuals in our sample were a random selection from the overall population, so we believe that the weighted averages are reflective of the population averages during the study period.

© 2024 Frenier C et al. *JAMA Health Forum*.

We report our results for continuous variables (age, credit score, medical debt in collections, non-medical debt in collections) as the mean and SD of the variable in each race/ethnicity sub-group for non-Hispanic White, Hispanic (all races), and Non-Hispanic Black enrollees. For binary variables (any medical debt in collections, any non-medical debt in collections, low credit score, sex, urban/rural residency), we report the weighted count and proportion of enrollees in each sub-group. We used a linear regression model estimated at the person-level to test for differences in means across the race/ethnicity groups. The p-values in Table 1 are taken from the coefficients on categorical variables for Hispanic and non-Hispanic Black indicators (reference = non-Hispanic White). The regression model for the financial health outcomes included control variables for age (linear and quadratic), sex, and parish of residence (fixed effects). The regression model for the testing for demographic differences was similar but omitted the controls for age, sex, and parish of residence. All models used standard errors clustered at the parish level.

Analyses were performed using R 4.1.1 and the analysis code will be archived here.

© 2024 Frenier C et al. *JAMA Health Forum*.
