# Supplementary material for: Financial Health Among Louisiana Medicaid Enrollees
Source: JAMA Health Forum. 2024 Oct 11;5(10):e243028. doi: 10.1001/jamahealthforum.2024.3028 (PMC11470384; doi:10.1001/jamahealthforum.2024.3028)
Supplement: Supplement 2. — Data Sharing Statement [file jamahealthforum-e243028-s002.pdf]

Data Sharing Statement

Frenier. Financial Health Among Louisiana Medicaid Enrollees. *JAMA Health Forum*.
Published October 11, 2024. doi:10.1001/jamahealthforum.2024.3028

Data
Data available: No

Additional Information
Explanation for why data not available: Data are not available due to restrictions imposed by the data use agreements with the Louisiana Department of Health and Equifax.
